# Population dynamics of mosquito species in a West Nile virus endemic area in Madagascar

**DOI:** 10.1051/parasite/2017005

**Published:** 2017-01-30

**Authors:** Luciano Michaël Tantely, Catherine Cêtre-Sossah, Tsiriniaina Rakotondranaivo, Eric Cardinale, Sébastien Boyer

**Affiliations:** 1 Laboratoire d’Entomologie Médicale, Institut Pasteur de Madagascar Ambatofotsikely Antananarivo 101 Madagascar; 2 Centre de coopération internationale en recherche agronomique pour le développement (CIRAD), UMR CMAEE 97491 Sainte Clotilde La Réunion France; 3 INRA, UMR 1309 CMAEE 34398 Montpellier France; 4 Centre de Recherche et de Veille sur les maladies émergentes dans l’Océan Indien (CRVOI) 97490 Sainte Clotilde La Réunion France

**Keywords:** Madagascar, Population dynamics, West Nile, *Aedeomyia madagascarica*, Trophic behavior

## Abstract

Human and animal serological surveys suggest that West Nile virus (WNV) circulation is widely distributed in Madagascar. However, there are no reported West Nile fever outbreaks or epizootics in the country and only one fatal human case has been reported to date. Currently there is very limited information on the maintenance and the transmission of WNV in Madagascar and particularly on the mosquito species involved in transmission cycles. In 2014, we initiated a study to investigate mosquito species composition, relative abundance, and trophic behavior in Mitsinjo District close to Lake Kinkony, a WNV endemic area in north-western Madagascar. We collected a total of 2519 adult mosquitoes belonging to 21 different species. The most abundant species was *Aedeomyia* (*Aedeomyia*) *madagascarica* Brunhes, Boussès & da Cunha Ramos, which made up 83% of all the mosquitoes collected. Mosquito abundance was associated with proximity to the lake (Morafeno and Ankelimitondrotra). Additionally, a correlation was observed between the lake-side biotope and the abundance of mosquito vectors in Morafeno. WNV RNA was detected in one pool of *Ae. madagascarica* and one pool of *Anopheles* (*Cellia*) *pauliani* Grjebine, suggesting that these two species may be involved in the maintenance and/or transmission of WNV in Madagascar.

## Abstract (fr)

Des études sérologiques humaines et animales suggèrent que la circulation du virus de la fièvre du Nil Occidental (VNO) est largement répandue à Madagascar. Toutefois, il n’existe pas de foyers de VNO ni d’épizooties dans le pays et seul un cas humain mortel a été signalé à ce jour. Actuellement, on dispose de très peu d’informations sur l’entretien et la transmission du VNO à Madagascar et en particulier sur les espèces de moustiques impliquées dans les cycles de transmission. En 2014, nous avons entrepris une étude pour étudier la composition des espèces de moustiques, l’abondance relative et le comportement trophique dans le district de Mitsinjo près du lac Kinkony, une zone endémique du VNO dans le nord-ouest de Madagascar. Nous avons recueilli un total de 2519 moustiques adultes appartenant à 21 espèces différentes. L'espèce la plus abondante était *Aedeomyia (Aedeomyia) madagascarica* Brunhes, Boussès & da Cunha Ramos, qui constituait 83 % de tous les moustiques collectés. L’abondance de moustiques était associée à la proximité du lac (Morafeno et Ankelimitondrotra). De plus, une corrélation a été observée entre le biotope bordure du lac et l’abondance de moustiques vecteurs à Morafeno. De l’ARN du VNO a été détecté dans un pool d’*Ae. madagascarica* et un pool d’*Anopheles* (*Cellia*) *pauliani* Grjebine suggérant que ces deux espèces peuvent être impliquées dans le maintien et/ou la transmission du VNO à Madagascar.

## 1. Introduction

West Nile virus (WNV) (family Flaviviridae, genus *Flavivirus*) was first isolated from a woman with febrile illness in the West Nile district of Uganda in 1937 [54]. The virus is transmitted to humans through the bite of a mosquito that has previously acquired the virus by blood-feeding on infected birds. The role of mosquito species in the WNV transmission cycle was first demonstrated in the species *Aedes albopictus* (Skuse) in 1943 [47]. The first isolations from human sera occurred in Egypt and Israel in 1951 [20, 23]. Subsequent events, including the emergence of WNV in North America in 1999, its spread westward across the United States, and then throughout the western hemisphere from South America to Canada, as well as repeated outbreaks in Europe [11, 12], suggest that WNV has the largest geographical distribution among the arthropod-borne viruses [24].

In Africa, WNV is endemic and widely distributed [45]. In South Africa, the first evidence of WNV infection was observed in 1958 [38]. Since this initial observation, large human epidemics due to changes in environmental conditions resulting in higher mosquito abundance [62] and high seroprevalence of WNV infection [26] have occurred in South Africa. WNV infections have also been detected in North Africa (Egypt, Tunisia, Algeria, Morocco, Senegal) [4, 9, 49, 52] as well as in Central Africa (Central African Republic, Kenya, Uganda, Nigeria) and Madagascar [4, 9, 14, 40, 52, 53]. Based on genetic differences, WNV strains have been classified in eight lineages, with lineages 1 and 2 being described as pathogenic. WNV Lineage 2 strains, which are endemic in sub-Saharan Africa and Madagascar, were previously considered to be of low pathogenicity [12, 30].

Mosquitoes of the genus *Culex* are the primary vectors of WNV in Africa and Asia due to their vector competence and host preferences. There is geographic variation within the genus due to the presence of locally important *Culex* (*Cx.*) species such as *Cx. pipiens pipiens* Linnaeus and *Cx. quinquefasciatus* Say in Nigeria [44]; *Cx. univittatus* Theobald in Kenya and South Africa; *Cx. theileri* Theobald in South Africa; and *Cx. neavei* Theobald, *Cx. quinquefasciatus* groups, and *Cx. poicilipes* (Theobald) in Senegal [3, 14]. In Asia, *Cx. quinquefasciatus*, *Cx. tritaeniorhynchus* Giles, and *Cx. vishnui* Theobald predominate [25]. Moreover, WNV vertical transmission has already been demonstrated in the field for several species: *Cx. univittatus*, *Cx. salinarius* Coquillett, *Cx. tarsalis* Coquillett, *Cx. erythrothorax* Dyar, *Cx. stigmatosoma* Dyar, and *Aedes triseriatus* (Say) [13, 39, 41, 42, 61] and experimentally for several other species: *Ae. aegypti* Linnaeus, *Ae. albopictus*, *Cx. pipiens*, *Cx. quinquefasciatus*, *Cx. tritaeniorhynchus*, and *Cx. modestus* Ficalbi [2, 3, 19, 34].

In Madagascar, virus isolation was first reported in 1978 from an endemic parrot species [10] and later from mosquitoes and humans [16, 17]. Despite serological and virological data demonstrating widespread circulation of WNV across the 18 districts of Madagascar that cover different bioclimatic zones of the country [16, 33, 36, 37, 43], neither epidemics nor epizootics of WNV have been reported to date. Only one lethal case due to WNV infection has been reported in a traveler returning from Madagascar in 2011 [31]. Among the 235 mosquito species described from the country [56], 29 species are widely associated with WNV infection, and they belong to five distinct genera: *Aedeomyia* (*Ad.*), *Aedes*, *Anopheles* (*An.*), *Culex*, and *Mansonia* [55]. Of these 29 mosquito species associated with WNV infection, 25 are not native to Madagascar [55, 56]: 12 species of genus *Culex*, 6 of *Aedes*, 4 of *Anopheles*, 1 of *Aedeomyia*, 1 of *Coquillettidia*, 1 of *Lutzia*, 2 of *Mimomyia*, and 1 of *Mansonia*. According to the proposed system of mosquito vector categorization that included natural infection, vector competence, and field vector-host contact, 4 of these 29 mosquito species were considered as major (*Culex quinquefasciatus*, *Culex tritaeniorhynchus*, *Culex univitattus*, and *Mansonia uniformis* (Theobald)), nine as candidate vectors (*Aedeomyia madagascarica*, *Aedes albocephalus* (Theobald), *Aedes circumluteolus* (Theobald), *Aedes aegypti*, *Aedes albopictus*, *Anopheles coustani* Laveran, *Culex antennatus* (Beker), *Culex decens* Theobald, and *Culex pipiens*) and the remaining (16 species) as potential vectors [55].

There have been few human and animal serological surveys of circulation rates of WNV in Madagascar in recent decades. In 1990, a first serological survey conducted in 12 regions of Madagascar reported a prevalence of 29.9% for anti-WNV antibodies in a non-random sample of 5–20 year-old children or young adults [43]. A second survey conducted in 1996 in children under 15 years of age in the highlands, and a third in 1999 along the north-western coast of Madagascar detected a 2.1% and 10.6% prevalence of anti-WNV antibodies, respectively [33]. Finally, in 2012–2013, a serological analysis of chicken sentinels was performed in areas close to lakes where domestic, wild, and migratory birds co-exist with humans and potential mosquito vectors were reported. This study revealed differences in the prevalence of anti-WNV antibodies between the two studied districts (Antsalova 29.4% and Mitsinjo 16.7%) [36].

These observations are consistent with several possible hypotheses regarding the persistence of enzootic/endemic WNV transmission in Madagascar. The first possibility is that there is a constant potential for contact between WNV vectors and humans due to the persistence of WNV in wild birds, regardless of the urban/rural setting. A second could be that there are alternating cycles in which urban and rural populations of vectors experience peaks in infection intensity separately, with each being the major contributor to WNV transmission at different times.

To gain a clearer understanding of the involvement of local mosquito populations as putative competent vectors in WNV transmission in Madagascar, a longitudinal entomological survey was carried out around Lake Kinkony. The lake is the second largest lake in Madagascar [28], supporting most of the wetland bird species of western Madagascar [35] with a strong vector density and a high WNV antibody prevalence [5, 36]. Given these conditions, it is an ideal location to understand the relationships between epizootic and putative sylvatic cycles. This preliminary study aims to determine population dynamics of mosquito species, along with their distribution, abundance, and feeding behavior in the WNV endemic context of Madagascar.

## 2. Materials and methods

### 2.1. Study sites

**Figure 1.**
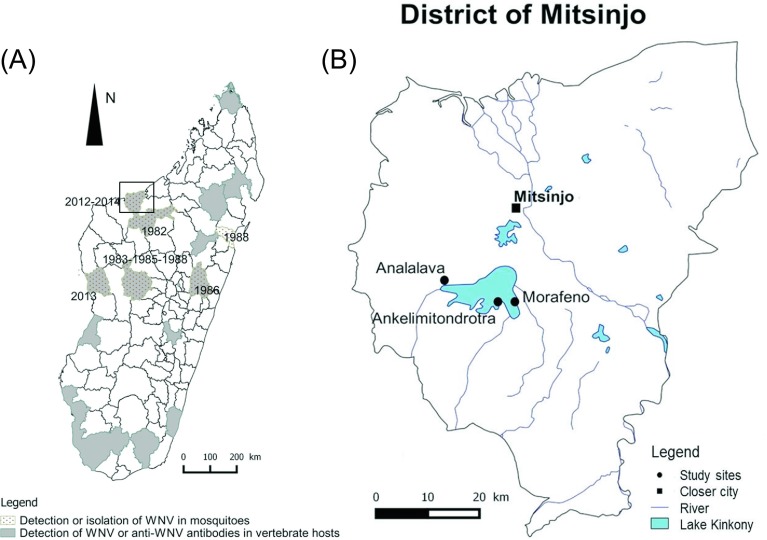
(A) Overall WNV detection in Madagascar (seroprevalence and virus identification) [16, 36, this study]. (B) Location of the study sites, part of the Mitsinjo district (February 2014 to December 2014).

In the western region of Madagascar, the district of Mitsinjo was chosen based on three criteria: (i) detection of a high WNV seroprevalence in domestic animals with WNV detection in mosquitoes in 2012, (ii) presence of migratory birds in the same area [36], and (iii) ecological features (lake, village, and forest) compatible with WNV transmission between domestic as well as wild wetland and forest birds. Three localities were investigated: Morafeno, Ankelimitondrotra, and Analalava forming an East-West transect across Lake Kinkony (Fig. 1). Morafeno, the most easterly village (16°08′74.6″ S, 45°55′17.5″ E), is surrounded by the lake, 100 m from the lakeside with a landscape made of jujube, tamarind dry forest, and food crops. Ankelimitondrotra (16°08′86.7″ S, 45°52′97.3″ E) is located on a peninsula-like projection into the lake. The village is located about 150–200 m from the lakeside with the landscape predominately composed of jujube and tamarind dry forest as well. A large area of swamp and marshland with aquatic plants is observed between Morafeno and Ankelimitondrotra as described by Andriamasimanana and Rabarimanana [1]. The village of Analalava, the most westerly village (16°08′35.7″ S, 45°42′02.5″ E), is located 1 km from the lakeside with a landscape consisting of jujube, mango, tamarind forest, and savannah dominated by Satrana palm. The average outside temperature is greater than 20 °C through the year and can exceed 25 °C during the wet season (September to May). The highest rainfall (200 mm–400 mm a year) was observed between January and March, while the lowest rainfall (<15 mm a year) is during the dry season (May and October) [8].

**Figure 2.**
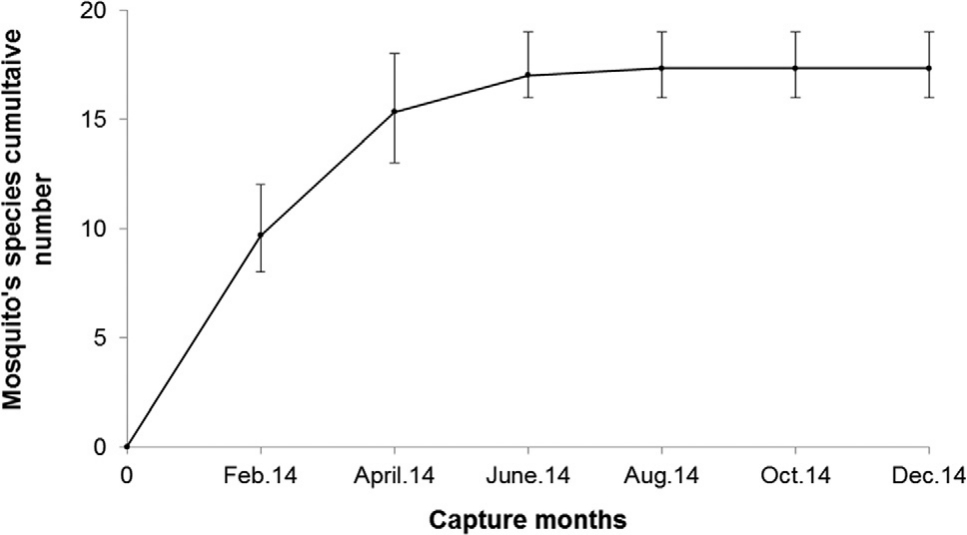
Mosquito species cumulative number for the 21 species caught around Lake Kinkony from February 2014 to December 2014. Standard errors bars indicate the standard deviations.

**Figure 3.**
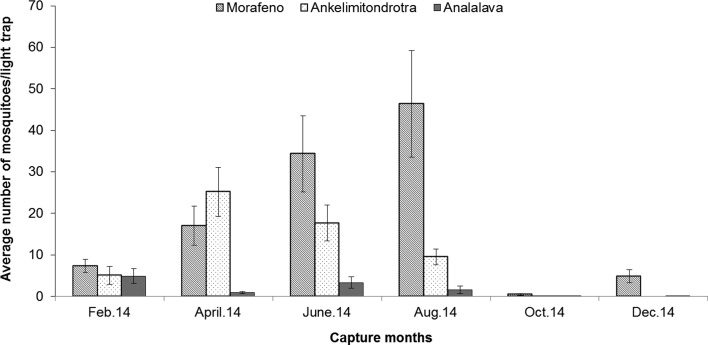
Distribution of all mosquitoes across the villages in the Lake Kinkony area (average number/CDC-light trap), standard error bars indicate standard deviations.

Fishing is the main occupational activity in Morafeno and Ankelimitondrotra; domestic animals (dogs) and livestock (cattle, sheep, goats, pigs, and poultry) are present in all three villages.

### 2.2. Longitudinal entomological survey

Mosquitoes were sampled every two months from February to December 2014 using CDC-light traps (CDC miniature light trap, BioQuip Products, Inc, Rancho Dominguez, USA) (12 in total) for the determination of mosquito population dynamics and poultry-baited BG sentinel traps (BGS traps) (BioQuip Products, USA) (five to six in total) positioned from 6:00 pm to 5:00 am. A longitudinal serological survey of chickens was performed concurrently. Both mosquito traps were distributed in three distinct ecosystems: forest-village transition zone, within village, and lakeside for one night of capture in each village (four light traps and two BG sentinels per ecosystem). The average distance between each ecosystem is about 100 m, except for Analalava where the distance between village and lakeside is 1 km. In August 2014, BG sentinel traps were not used in the village of Analalava due to security concerns.

Each mosquito specimen was morphologically identified by microscopy in the field and in the laboratory on a chilled table, after freezing in liquid nitrogen, based on morphological keys: the unpublished Fontenille key and the Brunhes key [6]. After identification, insects were pooled (1–10 individuals) per species, sex, and blood-feeding status of female (blood-engorged or not engorged), per trap and per zone. They were stored in liquid nitrogen in the field and stored at −80 °C in the laboratory. The host origin of the blood meals of 33 engorged mosquitoes collected in CDC-light traps was determined by the Beier method [4]. In all, seven vertebrate hosts were tested (human, rat, cow, pig, sheep, chicken, and dog) by a direct enzyme-linked immunosorbent assay (ELISA).

### 2.3. Screening mosquitoes for West Nile virus

The abdomens of 1825 unfed monospecific female mosquitoes were dissected, pooled, and ground up in 350 μL of Minimal Essential (MEM) cell culture medium (Gibco Life Technologies, USA) containing a mixture of 1000 U/mL penicillin, 1 mg/mL streptomycin, and 25 μg/mL amphotericin B (Sigma, USA) with two 3-mm diameter stainless steel beads (Loudet, France) for 30 s using the TissueLyser system (Qiagen, USA). Total RNA was extracted using the NucleoSpin RNA Virus kit (Macherey-Nagel, Germany). For WNV RNA detection, a capsid-based Taqman probe real-time PCR system able to detect WNV lineages 1 and 2 was used with AgPath-ID^TM^ One-Step RT-PCR Reagents (Ambion, Life Technologies, USA) [32] on a 7500 Real-time PCR system (Applied Biosystems, USA). A lineage 2 positive control was kindly provided by Dr S. Lecollinet (ANSES, France) and included in each of the tests for plate validation.

### 2.4. Statistical analysis

Data analyses were performed in R version 2.10.1 (R Foundation for Statistical Computing (http://www.r-project.org), and *p* ≤ 0.05 denoted statistical significance. The mosquito community structure at the different study sites and transects was analyzed using the following approaches: diversity using a Shannon equitability index (*H′*), and similarity of mosquito fauna using the Jaccard Index of similarity (*J*), based on the presence/absence of data only. We used the number of mosquito adults caught as the numeric surrogate for analyzing the effect of the types of trap, months, localities, and biotopes with a multi-way ANOVA (analysis of variance).We used Tukey’s honest significant difference (HSD) to determine which pairs of means were significantly different.

## 3. Results

### 3.1. Mosquito abundance, species diversity, and trophic preferences

**Figure 4.**
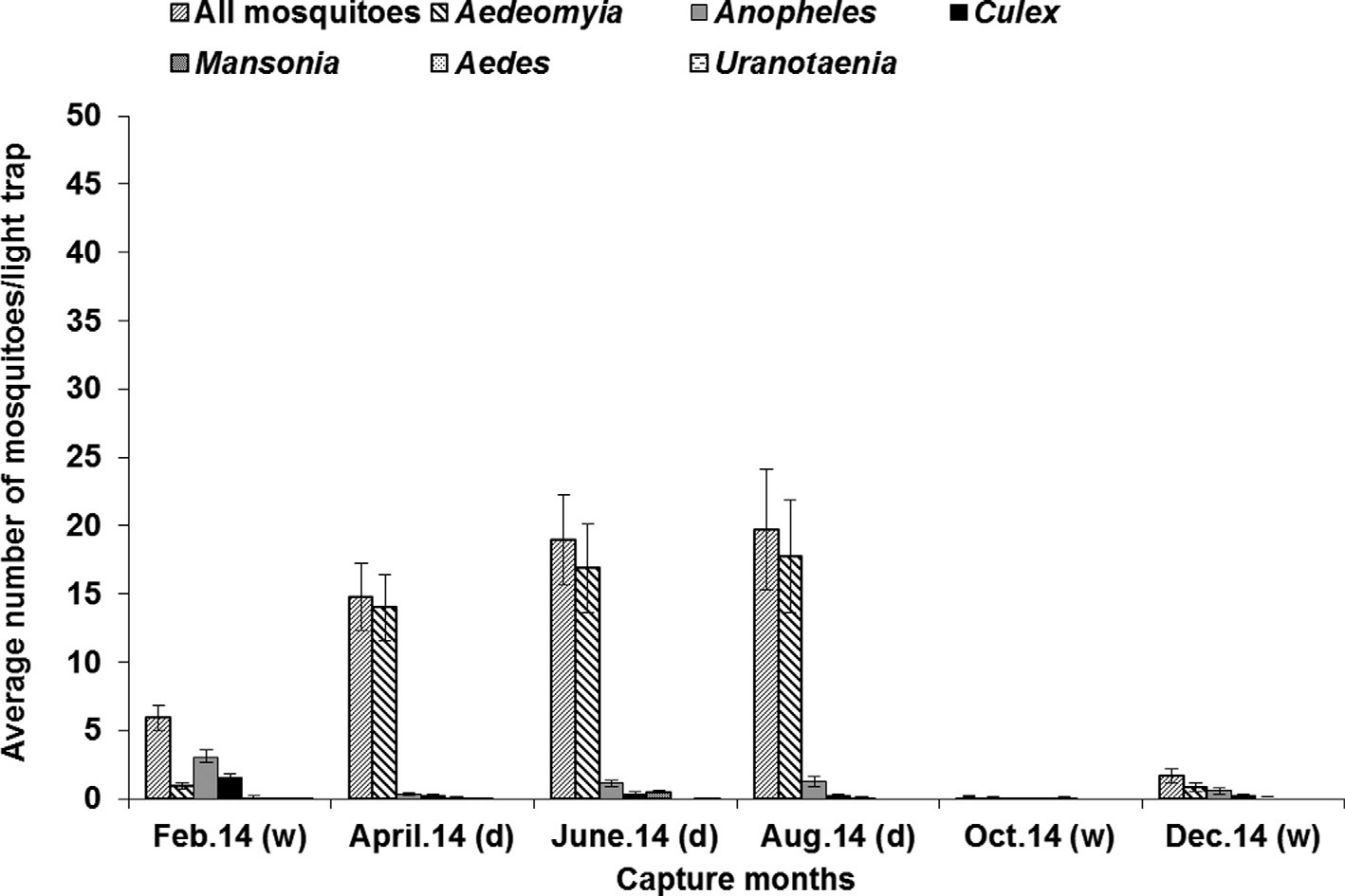
Average number of mosquitoes per light-trap in the Lake Kinkony area, data from the three villages being combined; standard error bars indicate standard deviations, d = dry season, w = wet season.

A total of 2519 specimens belonging to 21 mosquito species and 6 genera were identified (Tables 1 and 2). At each study site, no significant difference in terms of proportion of mosquitoes was observed between CDC-light traps and chicken-baited BG sentinel traps (Table 3; *df* = 1, *F* = 0.037, *p* > 0.8). When data from the three villages collected during six separate field samplings in a one-year period were combined, the species accumulation curve tended toward a plateau (Fig. 4). Two species of the genus *Aedeomyia* made up 82.33% of the adult catches. Ten and seven species of *Anopheles* and *Culex* genera accounted for 10.32% and 4.00% of the collection, respectively. The remaining, low-frequency, mosquito species captured (2.54%) consisted of species from the genera *Aedes*, *Mansonia*, and *Uranotaenia*. Greater species richness was found in Analalava (*H*′ = 2.94), followed by Morafeno (*H*′ = 2.77) and Ankelimitondrotra (*H*′ = 2.70). Similarity in species diversity was much higher (*J* = 0.82) between Morafeno and Ankelimitondrotra than between Morafeno and Analalava (*J* = 0.75), or between Ankelimitondrotra and Analalava (*J* = 0.61).

**Table 1.** Abundance of the 21 mosquito species collected at the adult stage between February 2014 and August 2014 in each village.

	Analalava			Ankelimitondrotra			Morafeno		
	BG	LT	Pools positive	BG	LT	Pools positive	BG	LT	Pools positive
*Aedeomyia furfurea* (Enderlein)	1	0	0/1	1	11	0/8	0	7	0/5
***Aedeomyia madagascarica*** Brunhes, Boussès & da Cunha Ramos*	0	17	0/4	31	595	0/57	252	1159	1/108
*Aedeomyia* sp.	0	0	0	0	10	0/1	0	0	0
*Aedes albodorsalis* Fontenille and Brunhes*	1	0	0/1	0	0	0	0	0	0
*Aedes argenteopunctatus* (Theobald)	0	0	0	0	1	0/1	0	0	0
*Aedes* sp.	5	1	0/3	1	0	0/1	0	0	0
***Anopheles coustani*** Laveran*	0	5	0/4	1	10	0/5	0	11	0/5
*Anopheles funestus* Giles*	0	0	0	0	2	0/2	1	5	0/3
*Anopheles gambiae* Giles*	2	26	0/7	2	2	0/4	2	13	0/11
*Anopheles maculipalpis* Giles	0	6	0/4	0	0	0	0	1	0/1
*Anopheles mascarensis* de Meillon	5	5	0/3	0	0	0	0	0	0
***Anopheles pauliani*** Grjebine*	15	1	0/2	1	1	0/2	2	37	1/13
*Anopheles pharoensis* Theobald*	0	7	0/1	0	26	0/4	3	30	0/7
*Anopheles squamosus/cydippis* *	4	12	0/5	0	4	0/1	4	12	3
*Anopheles rufipes* (Gough)	0	2	0/2	0	0	0	0	0	0
*Anopheles* sp.	0	0	0	1	1	0/1	0	2	1
***Culex antennatus*** (Becker)*	0	15	0/4	0	4	0/3	0	10	2
*Culex bitaeniorhyncus* Giles*	0	1	0/1	0	4	0/1	0	1	1
*Culex decens* Theobald	2	13	0/5	0	0	0	0	1	1
*Culex poicilipes* (Theobald)μ	2	0	0/1	1	4	0/4	0	14	1
*Culex tritaeniorhyncus* Giles*	3	2	0/3	0	3	0/1	0	11	5
*Culex univittatus* Theobald*	3	3	0/3	1	0	0/1	0	3	3
*Culex* sp.	1	0	0/1	1	0	0/1	1	3	2
***Mansonia uniformis*** (Theobald)*	14	12	0/7	2	12	0/6	7	5	5
*Uranotaenia* sp.	0	3	0/2	0	0	0	0	0	0
Total	58	131	0/64	43	690	0/104	272	1325	2/177

BG: Biogent sentinel. LT: light trap.

* Mosquito species collected in November 2012 in the same area [5].

Bold: mosquito species found naturally infected with WNV in Madagascar [16, 36].

μ Mosquito species found naturally infected with WNV in Africa [59].

**Table 2.** Abundance of the 21 mosquito species collected at the adult stage in the three biotopes (villages/forest/lakeside) for each village.

Species	Analalava			Ankelimitondrotra			Morafeno		
	Forest	Lakeside	Village	Forest	Lakeside	Village	Forest	Lakeside	Village
*Aedeomyia furfurea*	0	1	0	4	6	2	6	1	0
***Aedeomyia madagascarica*** *	2	6	9	246	209	171	326	582	503
*Aedeomyia* sp.	0	0	0	0	0	10	0	0	0
*Aedes albodorsalis* *	0	1	0	0	0	0	0	0	0
*Aedes argenteopunctatus*	0	0	0	0	0	1	0	0	0
*Aedes* sp.	1	0	5	0	1	0	0	0	0
***Anopheles coustani*** *	1	0	4	1	1	9	1	6	4
*Anopheles funestus* *	0	0	0	1	1	0	0	2	4
*Anopheles gambiae* *	1	1	26	1	3	0	3	4	8
*Anopheles maculipalpis*	1	0	5	0	0	0	0	1	0
*Anopheles mascarensis*	1	0	9	0	0	0	0	0	0
***Anopheles pauliani*** *	0	0	16	2	0	0	10	12	17
*Anopheles pharoensis* *	0	0	7	19	2	5	4	11	18
*Anopheles rufipes*	0	0	2	0	0	0	0	0	0
*Anopheles squamosus/cydippis* *	4	0	12	0	1	3	0	12	4
*Anopheles* sp.	0	0	0	0	1	1	0	1	1
***Culex antennatus*** *	7	1	7	1	2	1	0	6	4
*Culex bitaeniorhyncus* *	0	1	0	2	0	2	0	1	0
*Culex decens*	2	2	11	0	0	0	0	0	1
*Culex poicilipes* μ	0	2	0	1	3	1	0	9	5
*Culex tritaeniorhyncus* *	0	0	5	3	0	0	0	3	8
*Culex univittatus* *	2	0	4	0	0	1	0	0	3
*Culex* sp.	1	0	0	1	0	0	1	3	0
***Mansonia uniformis*** *	2	6	18	5	6	3	0	8	4
*Uranotaenia* sp.	0	1	2	0	0	0	0	0	0
Total	25	22	142	287	236	210	351	662	584

* Mosquito species collected in November 2012 in the same area [5].

Bold: mosquito species found naturally infected with WNV in Madagascar [16, 36].

μ Mosquito species found naturally infected with WNV in Africa [59].

**Table 3.** Analysis of variance to examine the effect of trap types (B), months (C), localities (D), and biotope nature (E) on the heterogeneity of mosquito species (A) in Lake Kinkony.

	*df*	Sum Sq	Mean Sq	*F* value	Pr (>*F*)
a-Combined localities					
A	20	0.0342	0.001709	2.612	0.00012***
B	1	0.0000	0.000024	0.037	0.84746
C	5	0.0014	0.000277	0.423	0.83288
D	2	0.0058	0.002910	4.448	0.01180*
E	2	0.0019	0.000948	1.449	0.23495
A × B	13	0.0011	0.000088	0.135	0.99989
A × C	45	0.0445	0.000988	1.510	0.01622*
A × D	29	0.0514	0.001772	2.709	2.50e−06***
A × E	29	0.1670	0.005759	8.804	<2.00e−16***
R	2293	1.5000	0.000654		
b-Morafeno					
A	16	0.0894	0.005588	5.607	7.09e−12***
C	5	0.0025	0.000490	0.492	0.78262
E	2	0.0033	0.001672	1.677	0.18723
A × C	22	0.0430	0.001956	1.962	0.00493**
A × E	17	0.3031	0.017832	17.892	<2e−16***
R	1505	1.5000	0.000997		
c-Ankelimitondrotra					
A	15	2.000e−29	1.600e−30	0.012	1.00000
C	4	4.920e−27	1.230e−27	8.796	6.34e−07***
E	2	3.000e−28	1.515e−28	1.084	0.33893
A × C	10	3.350e−27	3.349e−28	2.395	0.00852**
A × E	18	2.130e−27	1.185e−28	0.848	0.64330
R	670	9.367e−26	1.398e−28		
d-Analalava					
A	18	8.530e−30	4.739e−31	0.402	0.985
C	5	0.000e+00	1.000e−34	0.000	1.000
E	2	0.000e+00	0.000e+00	0.000	1.000
A × C	18	0.000e+00	0.000e+00	0.000	1.000
A × E	15	1.000e−32	6.000e−34	0.000	1.000
R	118	1.392e−28	1.179e−30		

× shows the effect of interaction between cited factors.

* *p* = 0.05;

** *p* = 0.01;

*** *p* = 0.001.

*df* = Degrees of freedom; Sum Sq = sum of squares; Mean Sq = mean of squares; *F* value = value of the *F* test; Pr (>F) = probability of the *F* test.

Mosquito abundance, driven mainly by the highly abundant species *Ad. madagascarica*, seemed to be associated with villages that are close to the lake (i.e. Morafeno and Ankelimitondrotra (Table 3; *df* = 2, *F* = 4.448, *p* < 0.01), with higher mosquito density observed in Morafeno (Tukey HSD tests: *p* < 0.001).

Correlation between lakeside biotope and the abundance of mosquito vectors was observed in Morafeno (Table 3; *df* = 17, *F* = 17.89, *p* < 0.001). Post hoc tests showed that this difference is only driven by the differences of *Anopheles* density between forest and lake (Tukey HSD tests: *p* < 0.001) and between forest and village (Tukey HSD tests: *p* < 0.001). No difference in mosquito density was observed for each of the mosquito species between lake and village in Morafeno (Tukey HSD tests: *p* > 0.05). No significant relationship between biotopes/ecotypes and mosquito abundance was observed in Ankelimitondrotra (Table 3; *df* = 18, *F* = 0.848, *p* > 0.64) and Analalava (Table 3; *df* = 15, *F* = 0.00, *p* = 1). We noted that in Ankelimitondrotra, the forest and lakeside ecosystems are located in close proximity, as the village is surrounded by the lake, while for Analalava, the lakeside biotope is located farther (approximately 1000 m) from the village and forest.

The highest and lowest abundance of caught adult mosquitoes was observed, respectively, in the village of Morafeno and the village of Analalava; with a high number of *Ad. madagascarica* obtained in Morafeno and Ankelimitondrotra (Table 3; *df* = 20, *F* = 2.61, *p* < 0.001). This species made up 81.54% (2054/2519) of the overall adult catches with light traps, being rare during the rainy season (November to March) and very abundant during the dry season (Fig. 2). The total amount of mosquitoes and *Ad. madagascarica* captured gave the same pattern of monthly variation when data from the three villages were combined (Fig. 3) (Table 3; *df* = 45, *F* = 1.51, *p* < 0.01). Whereas the peak of abundance of mosquitoes in Ankelimitondrotra was observed in April, the peak in Morafeno occurred in August with a sharp decline afterwards (Fig. 2).

Of the 33 blood meals analyzed from engorged mosquitoes, 15 (45%) of the total amount could not be identified due to the limits of the technique used. As shown in Table 4, most of the identifiable blood meals (11/18) were taken from domestic ruminants (cattle or sheep), six mixed blood meals were from cattle/sheep and one mixed blood meal from chicken/dog.

**Table 4.** Number of mosquitoes captured by poultry-baited BG sentinel traps and results from the blood meal analysis of engorged mosquitoes captured in light traps, including vertebrate host identification.

Genus	Capture with BG		nb. species tested	nb. pos/nb. tested	Antibody tested			
	nb. species	nb. adult			Sheep	Cattle	Cattle/Sheepβ	Poultry/Dogβ
*Aedeomyia* *	2	281	1*	1/1	0	0	0	1
*Aedes*	2	10	–	0/3	–	–	–	–
*Anopheles*	7	36	5μ	10/20	4	2	4	0
*Culex*	4	19	3°	7/7	1	4	2	0
*Mansonia*	1	24	–	0/1	–	–	–	–
*Uranotaenia*	1	1	–	0/1	–	–	–	–
Total	17	361	9		5	6	6	1

* *Aedeomyia madagascarica.*

μ *An. pauliani*: three blood meals from sheep and six mixed blood meals from cattle/sheep.

° *Cx. antennatus*: two blood meals from sheep and cattle.

β Mixed blood meal.

nb: stands for number.

### 3.2. Screening mosquitoes for West Nile virus

Eight mosquito species that have previously been found infected with WNV in Madagascar were collected during our study. Two of these species (*An. maculipalpis* and *Cx. decens*) were not collected in one of the localities (Ankelimitondrotra). When WNV genome detection was performed for a total of 346 abdomen pools representing 1825 mosquitoes (177 pools from Morafeno, 104 pools from Ankelimitondrotra, and 65 pools from Analalava), positive detection was observed in two of the tested pools: one pool of *Ad. madagascarica* (collected in April 2014) and one pool of *An. pauliani* (collected in August 2014). Both positive pools came from light traps placed near the lakeside in Morafeno.

## 4. Discussion

The species accumulation curve tending toward a plateau suggests that the number of species caught was approaching the total number of mosquito species in the area. Twenty-one mosquito species were collected in our study, a much larger number than the 14 species described in 2012 in the same area [5]. However, we did not collect seven mosquito species belonging to the *Aedes*, *Anopheles*, and the *Culex* genera that were collected near the site Morafeno in November 2012 [5]. This observation might be explained by the limitations of our sampling methods using only CDC-light traps and chicken-baited BG sentinel traps, in contrast to Boyer et al. (2014) in which other types of traps such as cattle-baited nets and backpack aspirators were used [5].

Of the eight mosquito species caught in our study area that were previously known to be infected by WNV in Madagascar [16, 36, 55], four were recently found WNV-positive in villages near lake areas: *Ad. madagascarica* and *An. coustani* near Lake Kinkony and *An. pauliani* and *Ma. uniformis* near Lake Soamalipo [36]. In our study, *Ad. madagascarica* and *An. pauliani* were the two species found to be positive for WNV. Unfed mosquito abdomens rather than whole specimens were screened for WNV infection. Detecting WNV in the abdomens of unfed mosquitoes suggests the potential for these two species to be involved in the maintenance and/or transmission of WNV in Madagascar. However, to incriminate these species as vectors of WNV transmission, vector competence studies should be undertaken to show infectious virus in the saliva [18, 48].

CDC-light traps are suitable for studying mosquito seasonal dynamics as evidenced by data obtained in Morocco [15], in sub-Saharan Africa [40], and in Madagascar [57, 58]. Interestingly, vector populations were abundant during the dry season with a great abundance of *Ad. madagascarica* (Fig. 3), contrasting with the findings in the central highlands of Madagascar, where WNV mosquito vectors and other arthropod-borne diseases are abundant at the beginning of and during the wet season [16, 57, 58]. Our results would suggest that the abundance of the potential vector *Ad. madagascarica* is influenced by temperature rather than by rainfall [51]. In Europe, abundance of WNV mosquito vectors was reported to be driven mostly by artificial flooding for human activities (cultivation, hunting, and fishing) rather than rainfall [2, 21].

This is the first time that *Ad. madagascarica* has been trapped in such large numbers in Madagascar, emphasizing its potential role in the WNV endemic cycle around the wetlands of Lake Kinkony. Indeed, species belonging to the genus *Aedeomyia* were rarely captured with other types of mosquito traps (light or baited traps) in Madagascar [5, 16, 46, 58]. Despite the lack of information on its larval stage biology, the abundance of this species at our study sites, mainly in Morafeno and Ankelimitondrotra, might be explained by the presence of the large swamp and marshland (with aquatic plants) that are favorable larval sites of the genus *Aedeomyia* [6].

Worldwide, there are seven species in the genus *Aedeomyia* [6, 22] with *Ad. africana* (absent from Madagascar) reported to be involved in the transmission of WNV in Africa [59]. In Madagascar, three species of the genus *Aedeomyia* have been collected (*Ad. madagascarica*, *Ad. pauliani*, and *Ad. furfurea*) [56] and previous detection of WNV in *Ad. madagascarica* on the island [36] has been confirmed by this study. This endemic species was recently described at adult stages that are morphologically close to those of *Ad. africana* [6].

Our results also raise questions as to how *An. pauliani* intervenes in WNV circulation around Lake Kinkony given its low relative abundance. Indeed, only a small number of this species were previously reported in the western wetlands (Lake Kinkony and Lake Soamalipo) of Madagascar [5]. However, this species might be an important WNV vector in a different biotope such as the village of Mampikony (district of Mampikony, 200 km east of lake Kinkony) where this species was found to be abundant [46]. *An. pauliani* is a general feeder [56], but is already considered as a potential vector of WNV due its rarity and to the lack of information on its vector competence [55].

Our results also support ornithophilic blood-feeding behavior, mainly for *Ad. madagascarica* in this study, in that chicken-baited BG sentinel traps proved to be very attractive for mosquitoes, thus confirming the use of poultry as an alternative to BG-Lure [5]. Indeed, BG sentinel traps were designed to collect anthropophilic mosquitoes by using artificial substances (BG-Lure) which are also present on human skin [29]. This attraction of *Ad. madagascarica* to poultry is in accordance with blood meal analysis, showing chickens as the blood meal source for this species (Table 4). The use of ELISA for blood meal analysis carries a risk of cross-reactivity but more notably fails to identify many potential hosts, including wild birds, as evidenced by the inability to identify more than 40% of blood meals from engorged mosquitoes. Further analysis using PCR-based methods targeting cytochrome B or cytochrome 1 genes [27] would provide confirmation of host blood meal sources. Unfortunately, at the time of the study, the technique was not routinely used in the laboratory. Our findings highlight the ornithophilic behavior already described for the genus *Aedeomyia* [6], and uphold the hypothesis of the involvement of ornithophilic mosquito species in the WNV epidemiological cycle [2, 7] and WNV circulation in bird populations in Madagascar [16, 17, 36]. The ornithophilic feeding preferences of *Ad. madagascarica* suggest this species might be involved in the maintenance of WNV in the mosquito/bird enzootic cycle, while other mosquito species considered as generalist feeders (Table 4) might serve as bridge vectors between birds and dead-end hosts (mammals) due to the low numbers of bird-to-bird feedings [60].

Finally, our results suggest that the abundance of mosquitoes around households, mainly *Ad. madagascarica*, depends on the distance between villages and lakes, suggesting mosquito heterogeneity in accordance with WNV incidence between villages around Lake Kinkony [36]. Although currently not documented in this study, more intense WNV transmission in villages near lakes would be expected, as highlighted by WNV detection in mosquitoes in Morafeno, given the correlation between lakeside ecotype and the abundance of *Ad. madagascarica* in this village. However, exposure of this species to a viremic host could not be excluded, given that WNV circulation was recently reported in this area [36]. For this reason, *Ad. madagascarica* is considered as a candidate vector of WNV due to the lack of information on its vector competence [55]. Vector competence studies need to be undertaken to conclude that this species may act as a bridge vector from wild to domestic birds, or *vice versa*, given that chickens, ducks, and geese are often left wandering during the night, facilitating bird-vector contact at our study sites. To demonstrate the vector competence of this species, the transmission of the virus during the feeding process is required with the determination of the dissemination rate throughout the mosquito body (legs, salivary glands) suggesting virus dissemination and transmission, which are known to depend on temperatures and viral loads [18, 48, 50]. An association with the presence of *Ad. madagascarica* in areas subject to WNV epizootics and/or epidemics could provide further evidence of its role in transmission.

In conclusion, our findings suggest that *Aedeomyia madagascarica* and *Anopheles pauliani* are potential vectors involved in the maintenance and/or transmission of WNV in Madagascar. Further work will provide greater knowledge of the host blood meals of these mosquitoes and better characterize the dynamics of WNV in this region, along with determining the WNV vector competence of these two species.
